# Influence of the hierarchical architecture of multi-core iron oxide nanoflowers on their magnetic properties

**DOI:** 10.1038/s41598-023-31294-4

**Published:** 2023-04-07

**Authors:** Stefan Neumann, Laura Kuger, Carsten-Rene Arlt, Matthias Franzreb, David Rafaja

**Affiliations:** 1 Institute of Materials Science, TU Bergakademie Freiberg, 09599 Freiberg, Germany; 2 Institute of Functional Interfaces, Karlsruhe Institute of Technology, 76344 Eggenstein-Leopoldshafen, Germany

**Keywords:** Magnetic properties and materials, Nanoparticles, Structural properties, Nanoscale materials, Transmission electron microscopy, Techniques and instrumentation, Characterization and analytical techniques, Imaging techniques, Microscopy, Materials science, Nanoscience and technology

## Abstract

Magnetic properties of superparamagnetic iron oxide nanoparticles are controlled mainly by their particle size and by their particle size distribution. Magnetic properties of multi-core iron oxide nanoparticles, often called iron oxide nanoflowers (IONFs), are additionally affected by the interaction of magnetic moments between neighboring cores. The knowledge about the hierarchical structure of IONFs is therefore essential for understanding the magnetic properties of IONFs. In this contribution, the architecture of multi-core IONFs was investigated using correlative multiscale transmission electron microscopy (TEM), X-ray diffraction and dynamic light scattering. The multiscale TEM measurements comprised low-resolution and high-resolution imaging as well as geometric phase analysis. The IONFs contained maghemite with the average chemical composition $\gamma$-Fe$_{2.72\pm 0.02}$O$_4$. The metallic vacancies located on the octahedral lattice sites of the spinel ferrite structure were partially ordered. Individual IONFs consisted of several cores showing frequently a specific crystallographic orientation relationship between direct neighbors. This oriented attachment may facilitate the magnetic alignment within the cores. Individual cores were composed of partially coherent nanocrystals having almost the same crystallographic orientation. The sizes of individual constituents revealed by the microstructure analysis were correlated with the magnetic particle sizes that were obtained from fitting the measured magnetization curve by the Langevin function.

## Introduction

In recent decades, magnetic iron oxide nanoparticles (IONPs) have emerged as one of the most promising nanomaterials for biomedical applications, for example as heat mediator for hyperthermia cancer treatment^1^, as carrier for drug delivery^2^ or as contrast agent in magnetic resonance imaging^3^. The manifold applications of IONPs arise from a combination of excellent properties including superparamagnetic behavior, high saturation magnetization, good biocompatibility and the possibility to functionalize IONPs by attaching various bioactive molecules.

IONPs usually consist of magnetite (Fe$_3$O$_4$) and/or maghemite ($\gamma$-Fe$_2$O$_3$), which crystallize in a spinel-like structure with tetrahedrally and octahedrally coordinated iron cations. Magnetite (space group $Fd{\bar{3}}m$) accommodates Fe$^{2+}$ and Fe$^{3+}$ cations on the Wyckoff positions 8*b* and 16*c*, respectively^4^. This distribution of the cations guarantees charge neutrality. However, in contrast to magnetite, some octahedral iron sites in maghemite must stay vacant to preserve the chemical composition Fe$_2$O$_3$ that corresponds to Fe$_{2.67}$O$_4$ in the spinel-like crystal structure. The oxygen sublattice is still fully occupied.

It has been shown that the Fe vacancies tend to order, which leads to the formation of different crystal structures of $\gamma$-Fe$_2$O$_3$. The crystal structure of $\gamma$-Fe$_2$O$_3$ with randomly distributed vacancies can still be described as a simple cubic spinel with the space group $Fd{\bar{3}}m$^5^. $\gamma$-Fe$_2$O$_3$ with vacancies partially ordered only on one of two distinct octahedral sites was described in the space group $P4_332$^6^, $\gamma$-Fe$_2$O$_3$ with vacancies partially ordered on one of three distinct octahedral sites in the tetragonal space group $P4_32_12$ but with almost identical lattice parameters *a* and *c*^7^. $\gamma$-Fe$_2$O$_3$ with fully ordered vacancies was described as a tetragonal superstructure in the space group $P4_12_12$ with $c\approx 3a$^8^. Vacancy ordering and the tetragonal distortion of the cubic spinel unit cell were originally reported for ‘microcrystalline’ $\gamma$-Fe$_2$O$_3$. However, the same phenomena were also observed in IONPs^9–11^.

The chemical composition (the [Fe]/[O] ratio) and related ordering of vacancies influence the magnetic properties of IONPs. They depend strongly on the fractions of Fe$_3$O$_4$ and $\gamma$-Fe$_2$O$_3$^12–14^, because Fe$_3$O$_4$ shows a higher saturation magnetization than $\gamma$-Fe$_2$O$_3$^15^. The size of IONPs is another important factor affecting their magnetic properties. When it decreases below a certain threshold value, IONPs become superparamagnetic^16^ as required for many biomedical applications^17–20^. The size threshold value is around 25 nm for Fe$_3$O$_4$ and 30 nm for $\gamma$-Fe$_2$O$_3$^21^. Therefore, the size of IONPs needs to be tailored for the respective application in order to obtain the best possible combination of properties. However, when IONPs are significantly smaller, their saturation magnetization is reduced by a disorder of the spins either in the interior of the IONPs or in their surface layer. The spin disorder in the interior of the IONPs was explained by inhomogeneous ordering of the cation vacancies^22^.The spin disorder in the surface layer of IONPs is usually explained by the incomplete coordination of superficial iron ions and the likely occurrence of structural defects at the IONP rim^23–25^. At 300 K, a thickness of the disordered spin layer of 0.54 nm was reported by Sharifi Dehsari et al.^26^, whereas a thickness of 1 nm was reported by Millan et al.^25^ (for IONPs larger than 3 nm).

Furthermore, the different [Fe]/[O] ratio in magnetite and maghemite is a reason for their different oxidation stability. Under aerobic conditions, maghemite is much more stable than magnetite^27^. Thus, the exact phase composition and distribution of Fe$_3$O$_4$ and $\gamma$-Fe$_2$O$_3$ can vary, in particular, if IONPs are in contact with oxygen. While a full oxidization of the iron oxide to $\gamma$-Fe$_2$O$_3$ was observed for smaller particles^28^, IONPs with intermediate sizes were found to contain non-stoichiometric Fe$_{\langle 3-\delta \rangle }$O$_4$ with $2.667<\langle 3-\delta \rangle <3$^12,28^. Large IONPs are generally assumed to have a core/shell structure with a Fe$_3$O$_4$ core and an oxidized shell^12,13,28–31^.

Recently, multi-core IONPs, often referred to as iron oxide nanoflowers (IONFs)^32–39^, have attracted attention of many research groups, as they show superior properties with respect to their mono-core counterparts, for instance a significantly enhanced specific loss power in magnetic hyperthermia^32–34^, but also increased cytotoxicity to cancer cells when applying an alternating magnetic field^36^. Lartigue et al.^33^ showed that the oriented attachment of individual cores building up the IONFs and the resulting continuity of their crystallographic orientation with a misalignment of the cores of only a few degrees^32,33,38–40^ favor a magnetic ordering across the interface and consequently a cooperative magnetic behavior. As a result, IONFs show enhanced magnetic susceptibility and smaller surface disorder^33^, while their superparamagnetic behavior is preserved^40^. Still, the magnetic performance of IONFs depends on many different properties such as the size of the cores, the size of the entire particles^33,38–42^, the number of the cores within the particles^39^ and their alignment^43^.

While a lot of research has been dedicated to the optimization of the synthesis process of IONFs^35,39^ and to the understanding of the magnetic interaction between individual cores within IONFs^44^, a profound description of the hierarchical structure of IONFs on the atomic scale, which is expected to influence the magnetic properties of IONFs significantly, has not been provided so far. In the present study, we describe the architecture and structure of IONFs on the nanoscopic and atomic scale, including crystallographic orientation relationships and structural coherence of the individual cores, and correlate these characteristics with the magnetic properties obtained from alternating gradient magnetometry (AGM) measurements. This contribution illustrates the capability of transmission electron microscopy (TEM) applied in high-resolution and low-resolution modes and networked by a correlative multiscale approach^45^, complemented by X-ray diffraction (XRD) and dynamic light scattering (DLS), to reveal detailed and statistically relevant information about the structure of IONFs on different length scales.

## Materials and methods

IONFs investigated in this study are commercially available dextran-coated maghemite IONFs (synomag-D, micromod Partikeltechnologie GmbH, Rostock, Germany) with a nominal hydrodynamic diameter of 50 nm, which were synthesized by a polyol method adapted from Lartigue et al.^33^. Details on the synthesis of the IONFs can be found in the paper from Gavilán et al.^35.^

For the TEM analysis, IONFs originally suspended in water were nebulized on a standard copper TEM grid covered with an amorphous carbon film. TEM experiments were carried out in a JEOL JEM-2200FS transmission electron microscope, which was equipped with a field emission gun operating at 200 kV, with a CESCOR probe aberration corrector (CEOS GmbH, Germany), with an ultra-high resolution objective lens ($C_S = 0.5$ mm), with an in-column energy filter ($\Omega$-filter) and with a highly sensitive 2k  $\times$ 2k  CCD camera (Gatan, Inc., USA). The $\Omega$-filter was used to remove inelastically scattered electrons from the beam and thus to improve the quality of the TEM images. The IONFs were characterized by high-resolution transmission electron microscopy (HRTEM), by scanning transmission electron microscopy (STEM) using an upper high-angle annular dark-field (HAADF) detector (EM-24630 UHADF, JEOL Ltd., Japan) and by selected area electron diffraction (SAED). Local diffraction patterns were obtained from HRTEM images using fast Fourier transform (FFT).

For XRD experiments, IONFs were dried in a fume hood and then spread on a ‘zero-background’ sample holder, which was a $\langle 5\,1\,0\rangle$-oriented Si single crystal. XRD measurements were carried out in symmetrical Bragg-Brentano geometry on a Seifert-FPM URD6 diffractometer (Freiberger Praezisionsmechanik, Germany) that was equipped with a sealed X-ray tube with a Cu anode, with a Soller collimator in the primary beam and with a graphite monochromator in the diffracted beam. The Soller collimator reduced the axial divergence of the primary beam. The graphite monochromator eliminated diffraction lines stemming from the spectral line CuK$_\beta$ and the fluorescence radiation of the sample. Measured XRD patterns were subjected to Rietveld refinement^46,47^ as implemented in the MAUD software^48^.

DLS experiments were carried out in backscatter mode using a ZetaSizer Nano ZS (Malvern Panalytical, UK). The laser wavelength was set to 632.8 nm, the detected scattering angle to $173^{\circ }$. In the DLS experiments, 100 $\upmu$L of IONF sample material ($c_{\text {IONF}}$ = 0.1 g/L) was injected into the capillary cell. The temperature (25 $^{\circ }$C) was controlled by the device. Due to the low IONF concentration, the viscosity of pure water at 25 $^{\circ }$C ($\eta _{\text {L}}$= 0.89 mPa·s) was assumed, when the results of the DLS experiments were evaluated.

AGM measurements were performed at room temperature in a gradient magnetic field that was generated by two magnetic coils. The maximum intensity of the external magnetic field ranged between $-4\cdot$10$^5$ A/m and $+4\cdot$10$^5$ A/m. The magnetic force induced by the external magnetic field was measured by a piezoelectric sensor. As the magnetic properties of the cores were of interest, the dextran shell of the IONFs was removed prior to the AGM measurements. In this preparation step, 300 $\upmu$L of a 25 g/L IONF suspension was mixed with 700 $\upmu$L pure ethanol and subsequently evaporated under stirring for 60 min at 95 $^{\circ }$C and at 300 min$^{-1}$. After evaporation, 1 mL pure ethanol was added in order to resuspend the dry IONFs. The suspension was stirred again at 300 min$^{-1}$ and 95 $^{\circ }$C for 60 min. After the second ethanol evaporation step, a dry, grey IONF powder was obtained. Approximately 1.5 to 3.0 mg of the powder was fixed between two adhesive films to produce a sample suitable for the AGM measurements. This sample was attached to a pendulum connected with the piezoelectric sensor. The measured magnetization curve was normalized to the sample mass and volume in order to determine characteristic magnetic values, i.e., the specific remanence and the specific saturation magnetization.

## Results

### Phase composition and vacancy ordering

**Figure 1:**
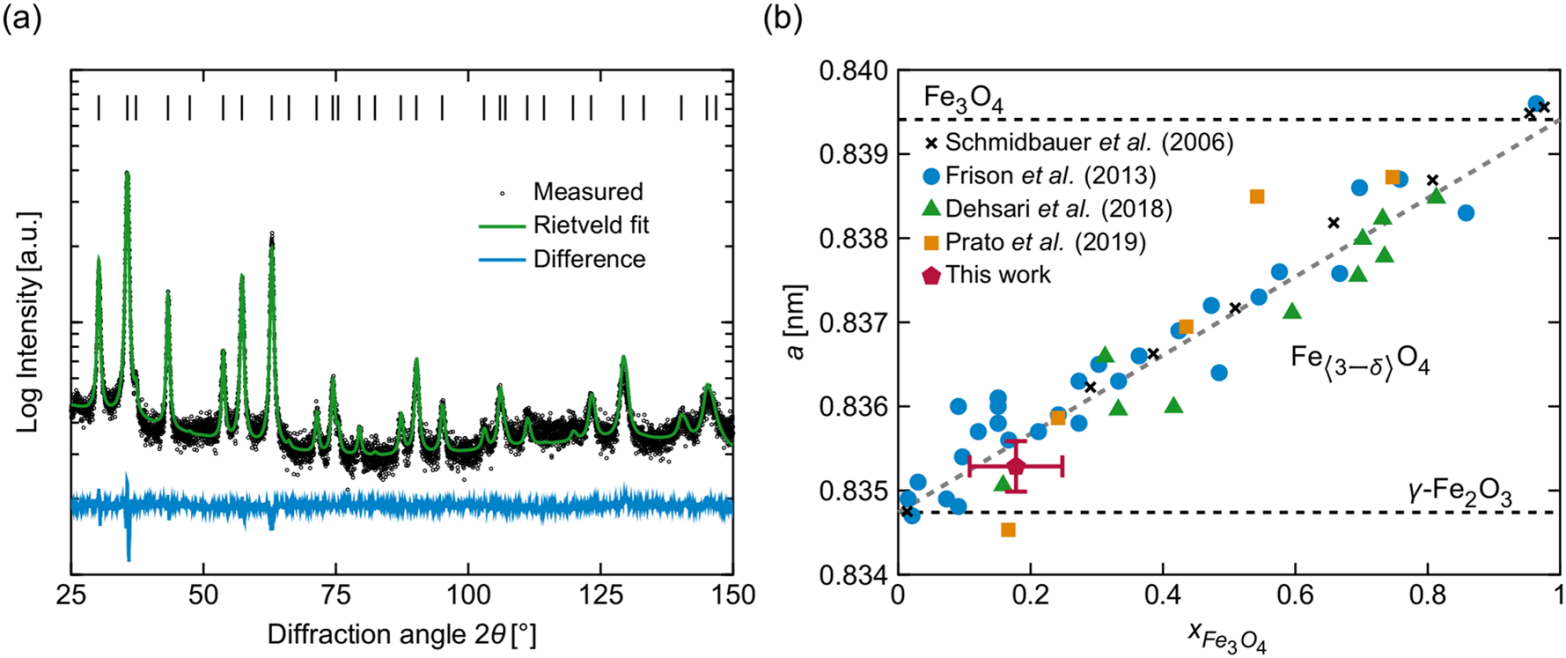
(**a**) XRD pattern of the IONFs under study. Rietveld refinement was carried out using space group $Fd{\bar{3}}m$. (**b**) Dependence of the cubic lattice parameter of IONPs on their stoichiometry. The horizontal dashed lines mark the lattice parameters of Fe_3_O_4_ and $\gamma$-Fe$_2$O$_3$. The Vegard dependence (ascending gray dashed line) was calculated for large crystallites ($D\rightarrow \infty$ in Eq. (1)). The black crosses^49^, blue circles^29^, green triangles^13^ and orange squares^14^ represent values taken from literature; the red pentagon with error bars marks the lattice parameter from the present work.

As mentioned in Introduction, the transition between Fe$_3$O$_4$ and $\gamma$-Fe$_2$O$_3$ is accompanied by a change in the oxidation state of the iron cations, which induces the formation and ordering of vacancies on the iron positions. Although the ordering of vacancies has to be described by different space groups ($Fd{\bar{3}}m$, $P4_332$, $P4_32_12$) from the crystallographic point of view^5–7^, the impact of vacancy ordering on the powder XRD pattern is rather weak^11,50^. The possible tetragonal distortion of the spinel-like cubic cell is small and thus hardly visible in powder XRD patterns, in particular in XRD patterns of NPs, which produce strongly broadened diffraction lines. Still, it has been demonstrated by many authors that the lattice parameter of IONPs with cubic or pseudo-cubic spinel structure depends linearly on the mole fraction of magnetite in maghemite^13,14,29,49^. Cervellino et al.^30^ extended this Vegard-like dependence to account for the effect of the crystallite size on the lattice parameter:

1 $$\begin{aligned} a = \bigl [(1-x_{\textrm{Fe}_3\textrm{O}_4})\cdot a_{\gamma \text{- }\textrm{Fe}_2\textrm{O}_3}+x_{\textrm{Fe}_3\textrm{O}_4}\cdot a_{\textrm{Fe}_3\textrm{O}_4}\bigr ](1-\Omega /D) \end{aligned}$$

In Eq. (1), $a_{\gamma \text{- }\textrm{Fe}_2\textrm{O}_3} = 0.83474$ nm^6^ and $a_{\textrm{Fe}_3\textrm{O}_4} = 0.83941$ nm^51^ are the terminal lattice parameters of maghemite and magnetite, respectively, $x_{\textrm{Fe}_3\textrm{O}_4}$ is the mole fraction of magnetite in maghemite, $\Omega$ is an empiric constant and *D* is the NP size. The ‘correction factor’ $(1-\Omega /D)$ describes the expansion of the lattice parameter in very small NPs, which results from surface relaxation effects^52–54^. Cervellino et al.^30^ determined $\Omega$ to be about $-2.05\times 10^{-3}$ nm. However, the effect of the NP size is apparent only for very small particles.

Rietveld analysis of the XRD pattern of the IONFs under study (Fig. 1a), which was carried out assuming a single-phase nature of the Fe$_{\langle 3-\delta \rangle }$O$_4$ sample and the space group $Fd{\bar{3}}m$, revealed the lattice parameter 0.8353(3) nm and a crystallite size of ($22\pm 3$) nm. In the Vegard-like dependence from Cervellino et al.^30^ (Fig. 1b), the refined lattice parameter (0.8353 nm) corresponds to the mole fraction $x_{\textrm{Fe}_3\textrm{O}_4}$ = 0.12(6) and to the stoichiometric coefficient $\langle 3-\delta \rangle$ = 2.71(2) of Fe$_{\langle 3-\delta \rangle }$O$_4$. Rietveld refinement of the site occupancy factors (SOFs) of the iron cations indicated that the majority of vacancies occurs on the octahedral sites 8*b* [SOF = 0.867(8)], while the tetrahedral sites 16*c* are almost fully occupied [SOF = 0.992(8)]. The oxygen anion sites 32*e* were assumed to be fully occupied [SOF = 1]. These SOFs correspond to the mole fraction $x_{\textrm{Fe}_3\textrm{O}_4}$ = 0.18(1) and to the stoichiometry $\langle 3-\delta \rangle =2.726(2)$ of Fe$_{\langle 3-\delta \rangle }$O$_4$. It should be mentioned that although iron vacancies are in general expected to occur exclusively on the octahedral sites^6,7,11^, Cooper et al.^50^ showed that in IONPs the number of tetrahedrally coordinated cation vacancies increases, when the particle size decreases below 8 nm.

**Figure 2:**
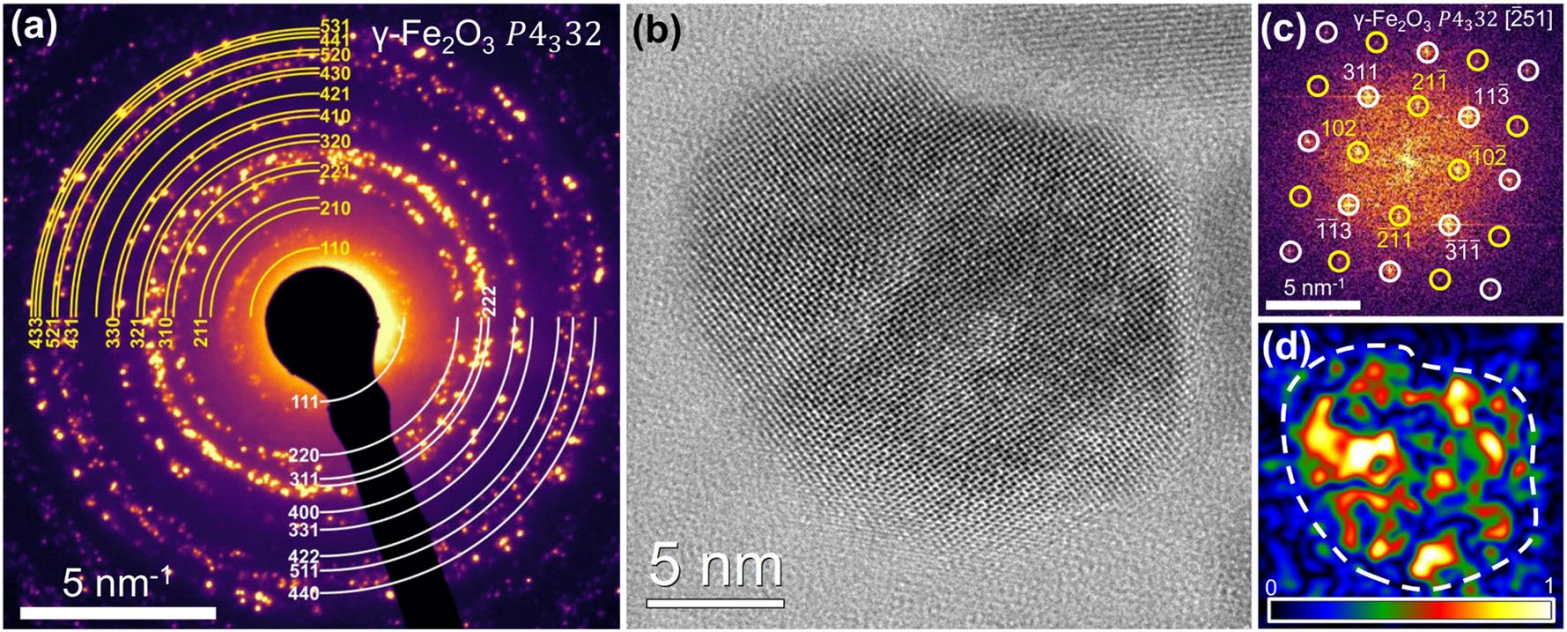
(**a**) SAED pattern of a large ensemble of IONFs. (**b**) HRTEM image of a single IONF core. (**c**) FFT of the HRTEM image shown in (**b**). (**d**) Amplitude image of the reflection 102. Diffraction patterns in (**a**) and (**c**) are indexed using space group $P4_332$. Reflections associated with vacancy ordering are marked in yellow.

The SAED pattern (Fig. 2a) and the FFT (Fig. 2c) of the HRTEM image (Fig. 2b) show superstructure reflections (marked in yellow). Their presence indicates that the vacancies in the IONFs are ordered to a certain extent, as it would correspond, e.g., to the space group $P4_332$. In order to rule out a tetragonal distortion of the cubic unit cell, which was reported by Jørgensen et al.^9^ and Andersen et al.^11^ for IONPs with ordered vacancies, the XRD pattern from Fig. 1a was alternatively refined using the tetragonal space group $P4_32_12$. However, this Rietveld refinement revealed the same lattice parameters $a = c = 0.8353(5)$ nm, as no noticeable tetragonal distortion was observed.

In order to find out, whether the vacancies are ordered throughout the whole particle or just locally, the amplitude images of the lattice fringes $\{1\,0\,2\}$ obtained from geometric phase analysis (GPA)^55,56^ were taken into consideration. As the lattice fringes $\{1\,0\,2\}$ only appear in crystal structures with ordered vacancies (space group $P4_332$ or $P4_32_12$), the magnitude of the local amplitudes obtained from GPA is a measure of the amount of ordered vacant octahedral positions. In the amplitude image (Fig. 2d), bright colors correspond to a higher amount of ordered vacancies, dark colors to a lower amount of ordered vacancies. A highly non-homogeneous distribution of ordered vacancies is apparent. Complementarily to the results of XRD, which proved that the IONFs under study are almost entirely oxidized to maghemite (cf. Fig. 1b), the amplitude image from Fig. 2d shows that the vacancies are ordered only in few regions, which form subdomains with a size of few nanometers.

### Arrangement and coherence of individual cores in the IONFs

**Figure 3:**
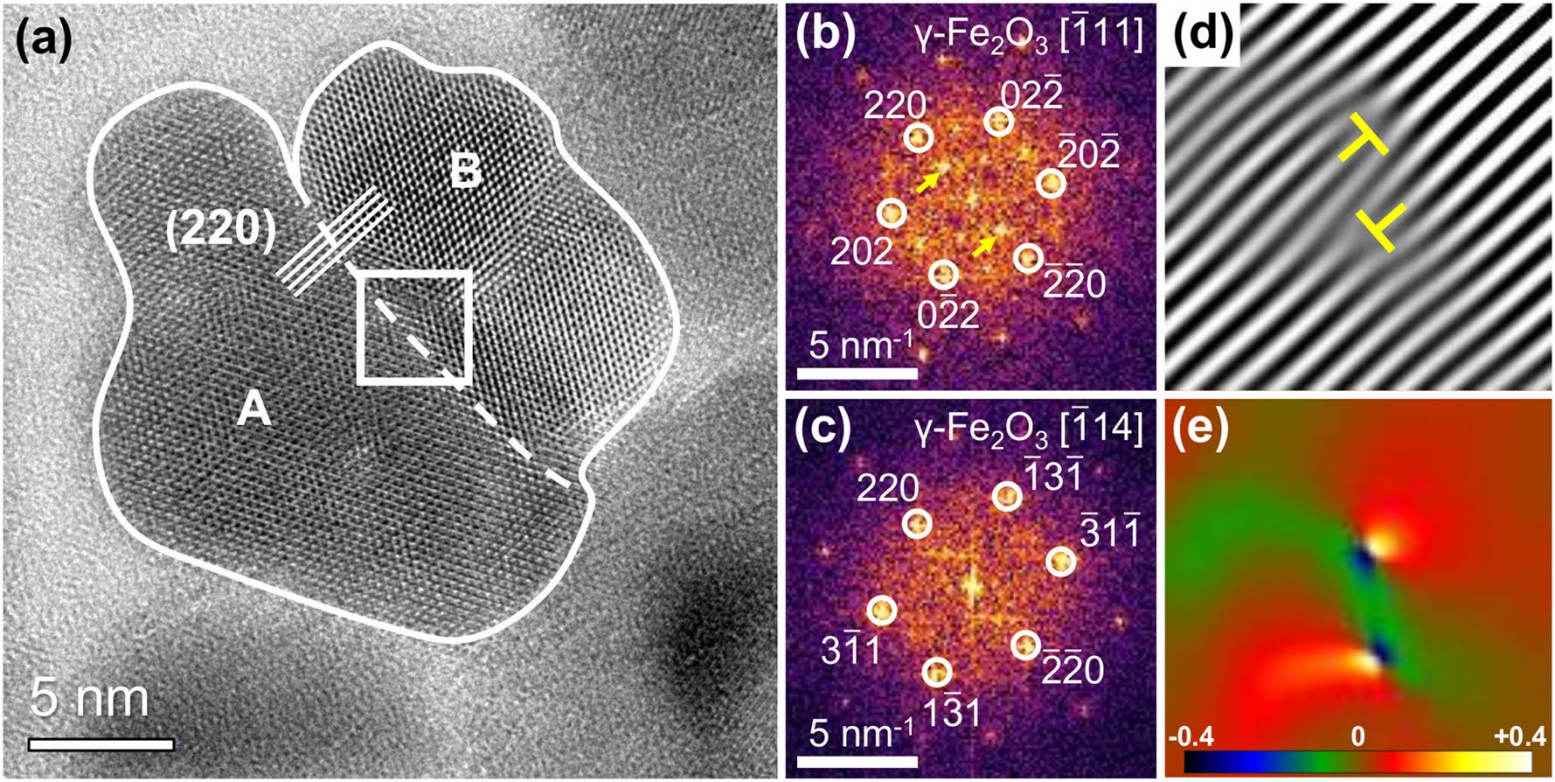
(**a**) HRTEM image of a double-core IONF. The outer boundaries of the individual cores and their interface are indicated by a solid line and by a dashed line, respectively. Panels (**b**) and (**c**) show local FFTs of the cores labeled A and B in (**a**), respectively. In panel (**b**), reflections associated with the ordering of vacancies are marked by arrows. (**d**) Filtered inverse FFT showing the $(2\,2\,0)$ lattice fringes from the region in the middle of panel (a) that is marked by a square. (**e**) Strain field component perpendicular to the $(2\,2\,0)$ lattice planes of the cores as determined by GPA.

Although separated cores were found occasionally for the IONFs under study (Fig. 2b), the majority of IONFs consists of agglomerated cores (Fig. 3). Several authors reported that individual cores within IONFs tend to have the same crystallographic orientation^32,33,35,43^. The cores in the IONFs under study possess distinct crystallographic orientation relationships, but the majority of them was mutually twisted. The IONF in Fig. 3a is composed of two cores, which are attached along their lattice planes $(2\,2\,0)$ and mutually twisted by about $35.3^{\circ }$ around the crystallographic direction $[1\,1\,0]$. The twist angle was determined from the angle between the crystallographic directions $[{\bar{1}}\,1\,1]$ and $[{\bar{1}}\,1\,4]$, which were assigned to the direction of the primary electron beam for the core A and B, respectively (Fig. 3b and c). Note that the angle of $35.3^{\circ }$ corresponds to the smallest angle between the crystallographic directions $\langle 1\,1\,1\rangle$ and $\langle 1\,1\,4\rangle$. The filtered inverse FFT image showing strongly magnified $(2\,2\,0)$ lattice fringes (Fig. 3d) reveals some discontinuities at the interface of the cores, which resemble dislocations. The presence of these crystal structure defects is confirmed by the strain field component perpendicular to the $(2\,2\,0)$ lattice planes of the cores (Fig. 3e), which corresponds to the strain distribution that is typically observed around the cores of edge dislocations^56,57^.

**Figure 4:**
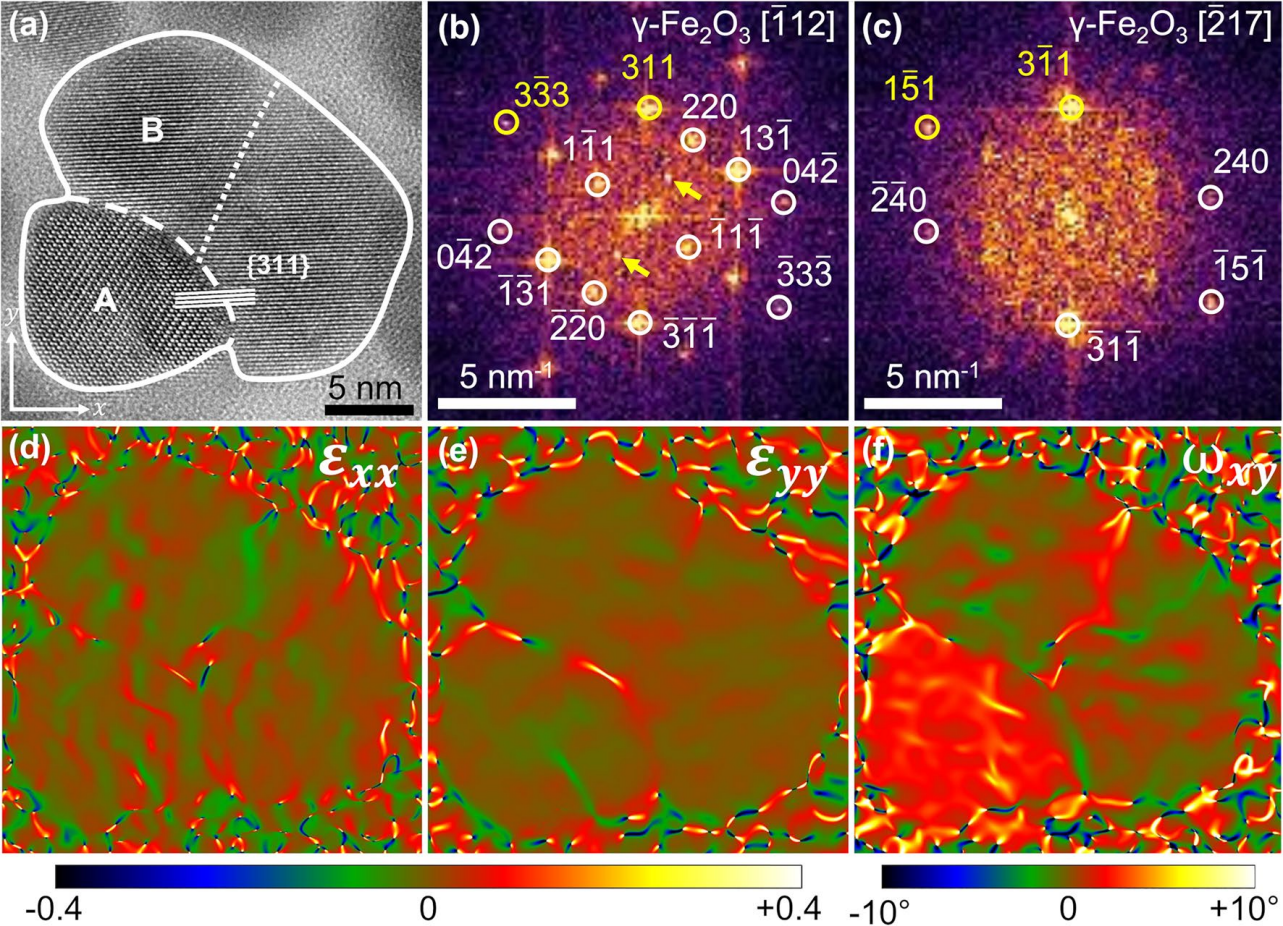
(**a**) HRTEM image of a double-core IONF. The outline of the IONF, the interface between the two cores, and the interface between individual nanocrystals within the larger core are indicated by a solid, dashed and dotted line, respectively. Panels (**b**) and (**c**) show local FFTs of the cores labeled A and B in (**a**), respectively. The spots marked by yellow circles were used for GPA. Reflections associated with the ordering of vacancies are marked by arrows in (**b**). The strain field components $\varepsilon _{xx}$ and $\varepsilon _{yy}$ and the rigid rotation field $\omega _{xy}$ determined by GPA are shown in panels (**d**), (**e**) and (**f**), respectively. The coordinate system is provided in the lower left corner of panel (**a**).

Another double-core IONF is depicted in Fig. 4a. Also in this case, individual cores possess a specific orientation relationship. They share the lattice planes $\{3\,1\,1\}$ and are mutually twisted by about $19.2^{\circ }$, which is the angle between the crystallographic directions $[{\bar{1}}\,1\,2]$ and $[{\bar{2}}\,1\,7]$ (cf. Fig. 4b,c). Moreover, these cores share additional lattice planes, e.g., $(0\,4\,{\bar{2}})_{\text {A}} \parallel (2\,4\,0)_{\text {B}}$, $(0\,{\bar{4}}\,2)_{\text {A}} \parallel ({\bar{2}}\,{\bar{4}}\,0)_{\text {B}}$, $(3\,{\bar{3}}\,3)_{\text {A}} \parallel (1\,{\bar{5}}\,1)_{\text {B}}$ or $({\bar{3}}\,3\,{\bar{3}})_{\text {A}} \parallel ({\bar{1}}\,5\,{\bar{1}})_{\text {B}}$. Note that the lattice planes $\{3\,3\,3\}$ and $\{5\,1\,1\}$ have the same interplanar spacing in cubic structures. The coincidence of several lattice planes is a possible reason for the shape of the interface between the individual cores. In contrast to the straight interface between the cores from Fig. 3, which is more or less perpendicular to the shared lattice planes $(2\,2\,0)$, the interface between the cores in Fig. 4 is rather curved, because its direction is not restricted by a single coinciding family of lattice planes.

A more detailed information about the local misorientations of the cores was obtained from GPA^55,56^ that was performed on the ‘non-colinear’ reflection spots $3\,1\,1_\text {A}\parallel 3\,{\bar{1}}\,1_\text {B}$ and $3\,{\bar{3}}\,3_\text {A}\parallel 1\,{\bar{5}}\,1_\text {B}$. The strain field components $\varepsilon _{xx}$ and $\varepsilon _{yy}$ shown in Fig. 4d,e, which represent the strain parallel and perpendicular to the $\{3\,1\,1\}$ lattice planes of the cores, reveal that the lattice strain is primarily concentrated at the interface of the cores, whereas no apparent strain seems to be present within the cores. The rigid rotation field $\omega _{xy}$ shown in Fig. 4f disclosed that the cores A and B are additionally twisted along the viewing direction by about $2^{\circ }$. Moreover, Fig. 4f suggests that core B is further fragmented into smaller nanocrystals (NCs) that are slightly twisted with respect to each other along the viewing direction by about $0.3^{\circ }$. Thus, the size of the primary building blocks within the IONFs is actually smaller than 10 nm.

**Figure 5:**
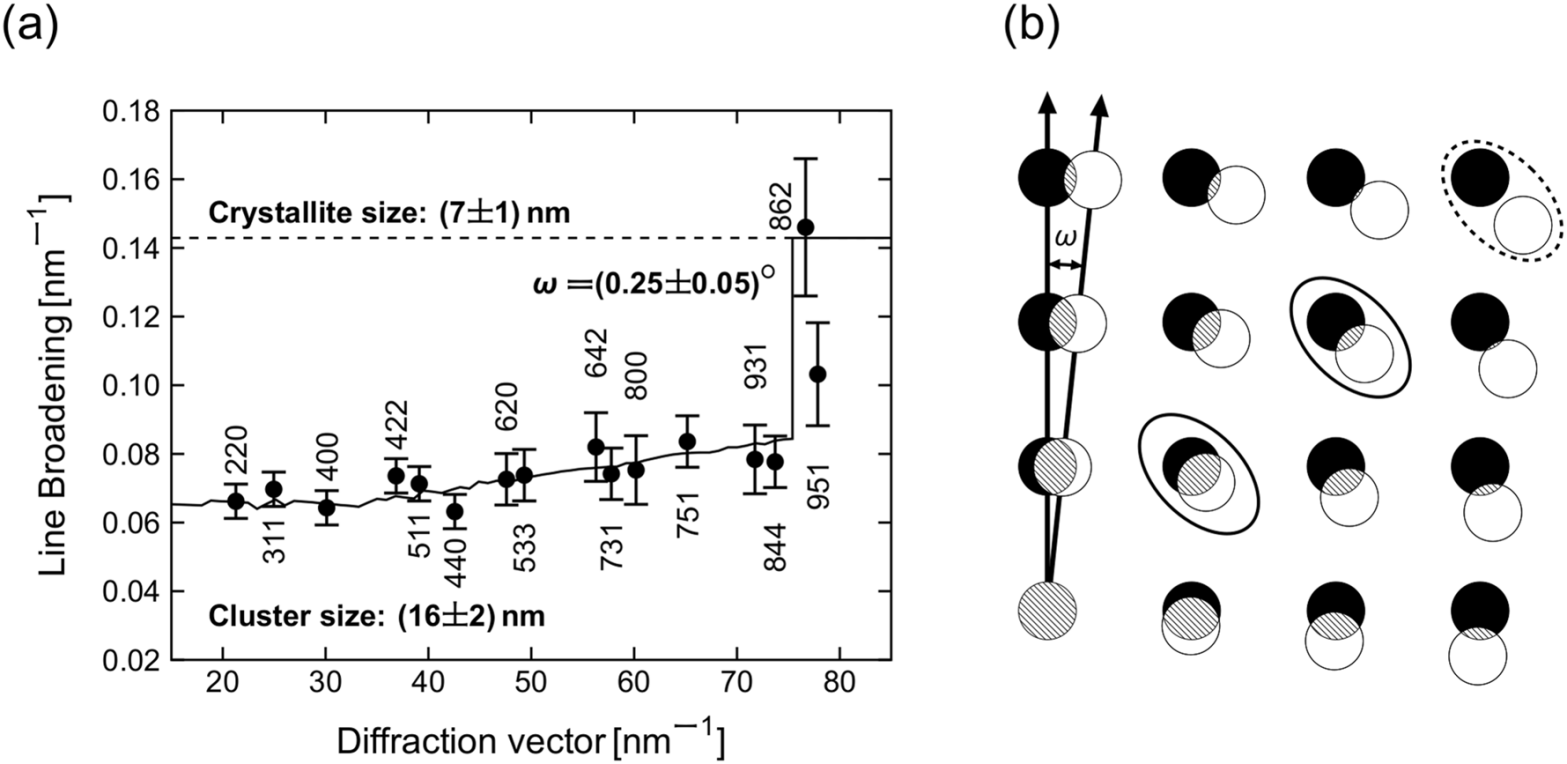
(**a**) Dependence of the XRD line broadening expressed in the reciprocal space units, ${\text {FWHM}}({\text {rad}}) \cdot \cos \theta /\lambda$, on the magnitude of the diffraction vector, $|\textbf{q}| \equiv q = 4\pi \sin \theta / \lambda$. Black circles represent experimental data, the black solid line shows the dependence of the line broadening on $|\textbf{q}|$ calculated for partially coherent NCs according to Rafaja et al.^58^. (**b**) Schematic representation of the effect of the mutual misorientation of crystallites by the angle $\omega$ in direct space on the rotation of their reciprocal lattices, adapted from Rafaja et al.^59^. The reciprocal lattice points of two different crystallites are shown by filled and empty circles, respectively. The overlap of the reciprocal lattice points (hatched areas) represents the degree of partial coherence of the crystallites that decreases with their increasing distance from the origin of the reciprocal lattice^58,59^. Solid ellipses mark two examples of overlapping pairs of reciprocal lattice points. The dashed ellipse marks separated (non-coherent) reciprocal lattice points.

The fragmentation of the IONF cores was confirmed by XRD. The XRD line broadening that was obtained by fitting individual XRD lines with Pearson VII functions^60,61^ increased steeply at $|\textbf{q}| \approx 75\,\textrm{nm}^{-1}$ (Fig. 5a), which is an indicator of the partial crystallographic coherence of adjacent NCs^58,59^. In previous reports^58,59^, it was shown that adjacent crystallites can be partially coherent for XRD, if they are sufficiently small and if they possess very similar crystallographic orientations. Such crystallites cannot be distinguished by XRD from each other and appear larger. The degree of the partial coherence corresponds to the volume of the overlapping parts of the reciprocal lattice points (Fig. 5b), which depends on the size of the reciprocal lattice points (approx. reciprocal value of the size of individual NCs), on the misorientation of neighboring NCs ($\omega$) and on the magnitude of the diffraction vector. A consequence of the partial coherence of NCs is a ‘narrowing’ of the XRD lines that appears at short diffraction vectors. The dependence from Fig. 5a was described by a model from Rafaja et al.^58^. The refinable parameters of the model were the size of the crystallites and their local misorientation. The cluster size corresponds to the reciprocal value of the XRD line broadening extrapolated to $|\textbf{q}| = 0$. The refinement revealed a cluster size of 16 nm, a primary crystallite size of 7 nm and a crystallite misorientation of $0.25^{\circ }$. The cluster size, the crystallite size and the misorientation of crystallites agree very well with the parameters determined from HRTEM and GPA (cf. Fig. 4).

### Statistical determination of particle, core and shell size

The results of HRTEM and XRD experiments discussed above confirmed that the majority of IONFs under study consists of agglomerates of nanocrystalline cores having specific mutual crystallographic orientations. However, these techniques cannot reveal statistically reliable information about the size distribution of the respective objects. HRTEM is typically applied to image few particles, thus its statistical reliability is low. XRD probes a significantly larger volume of the sample. However, the crystallite size distribution is usually obtained from the shape of the XRD lines assuming a certain shape of the distribution function^62^. This approach is not easily applicable for partially coherent NCs, because the partial coherence of adjacent NCs affects the shape of the XRD lines in addition to the size distribution and microstrain (variation of the interplanar spacing)^63^.

**Figure 6:**
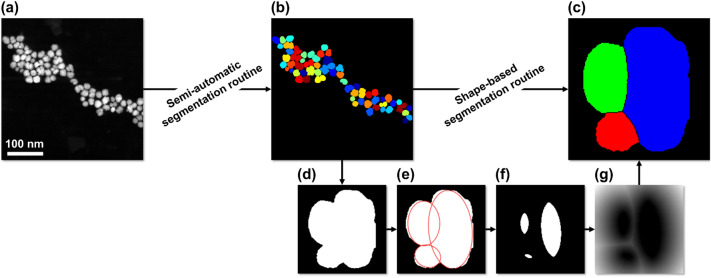
Schematic representation of the multi-stage segmentation routine used for the determination of the particle size and core size distribution. (**a**) Original low-magnification HAADF-STEM image of the IONFs. (**b**) HAADF-STEM image segmented into individual particles by the semi-automatic segmentation routine from Neumann et al.^45^. (**c**) Single IONF segmented into several cores by a shape-based segmentation routine. (**d**) Binary image of a single segmented IONF. (e) Shape of the IONF and its individual cores approximated by ellipses based on the DTECMA algorithm^64^. (**f**) Shape markers determined on the basis of the ellipses from (**e**). (**g**) Outer Euclidean distance transform of the shape markers from (**f**) used as the marking function for the watershed segmentation of the IONF into its cores.

In order to gain statistical insights into the size distribution of the entire IONFs and the individual cores, low-magnification HAADF-STEM imaging was employed. This technique allows to visualize 50-100 particles in a single low-magnification HAADF-STEM image. The HAADF-STEM images were evaluated using a multi-stage segmentation routine based on the watershed algorithm^65^. In the first stage of the routine, accumulated IONFs (Fig. 6a) were segmented into individual particles (Fig. 6b) by a semi-automatic segmentation routine^45,66^. For this segmentation step, the image intensity was adjusted, the noise was reduced using a Gaussian filter, the pre-processed images were binarized and morphologically smoothed^45^. Finally, individual particles were segmented using a marker-based watershed transformation. The markers were determined based on the extended minima transform of the inverted inner Euclidean distance transform of the pre-processed binary image^67^. The result of the segmentation routine was inspected and critical regions of the image were segmented manually. From the segmented images (Fig. 6b), the area-equivalent diameter $d_A$ of individual IONFs was determined using

2 $$\begin{aligned} d_A = \sqrt{\frac{4A}{\pi }} \end{aligned}$$

where *A* is the area of the IONFs.

In the second step of the multi-stage segmentation routine, every individual IONF was segmented into its cores by a segmentation routine that considers mainly the IONF shape (Fig. 6c). When an IONF consists of coalesced cores, its contour shows concave points (Fig. 6d). Individual cores were localized using the Distance Transform-based Ellipse Contour Matching Algorithm (DTECMA)^64^ that was applied to binary images of individual IONFs (Fig. 6e). This algorithm identifies overlapping objects—in this case individual cores of an IONF—by approximating their two-dimensional projections with ellipses. Afterwards, shape markers were determined based on the extended minima transform of the inverted inner Euclidean distance transform^67^ of the binary images of the individual ellipses determined by the DTECMA algorithm (Fig. 6f). Finally, the outer Euclidean distance transform of the shape markers (Fig. 6g) was determined and used as the marking function for the watershed segmentation of the IONFs into their cores. The segmentation of the IONFs into their cores was controlled by adjusting the parameters of the DTECMA algorithm, i.e., the distance threshold influencing the extraction of concave points and the regularization parameter balancing the number of ellipses, as well as by adjusting the threshold value of the extended minima transform that was used to determine the shape markers. The size of the individual cores was then determined analogously to the size of the IONFs (Eq. 2).

**Figure 7:**
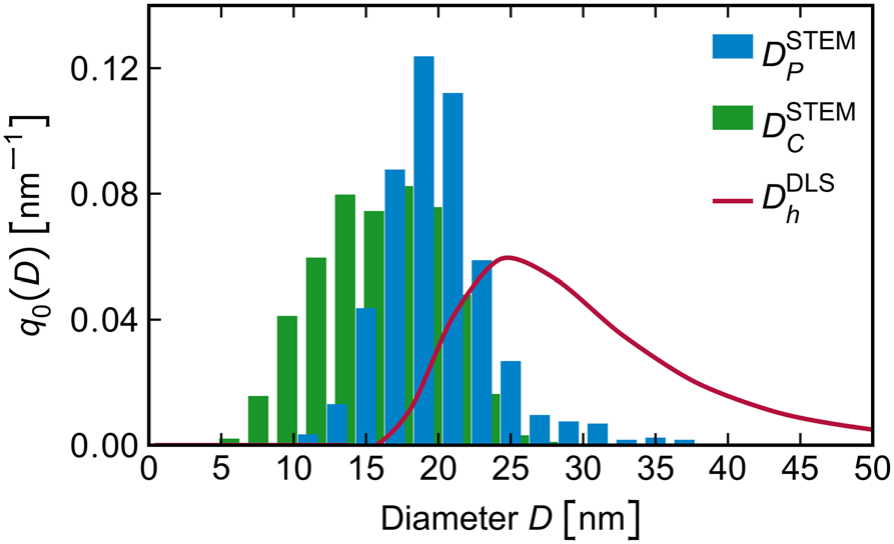
Number distribution density ($q_0$) of the size of the IONFs ($D_P^{\text {STEM}}$), their cores ($D_C^{\text {STEM}}$) and the hydrodynamic diameter ($D_h^{\text {DLS}}$) as determined using HAADF-STEM and DLS, respectively.

The size distribution of the IONFs and the individual cores determined from HAADF-STEM images using the multi-stage segmentation routine are depicted in Fig. 7 together with the size distribution of the hydrodynamic diameter of the IONFs that was determined using DLS. In order to be able to compare the size distribution determined using DLS with the size distributions derived from HAADF-STEM images, the intensity distribution density $q_{6}(D_h)$ that is primarily provided by DLS must be converted to the number distribution density $q_{0}(D_h)$ using^68^

3 $$\begin{aligned} q_{0}(D_h) = \frac{D_h^{-6}q_{6}(D_h)}{\int _{D_{h,\text {min}}}^{D_{h,\text {max}}}D_h^{-6}q_{6}(D_h)\text {d}D_h} \end{aligned}$$

Note that the hydrodynamic diameter of the IONFs corresponds to their size including the dextran shell. As HAADF-STEM imaging uses electrons scattered by atomic nuclei to high angles, it is highly sensitive to the atomic number of the scattering atoms^69^. For this reason, HAADF-STEM imaging visualizes IONFs almost without their light dextran shell. Moreover, the dextran shell degrades quickly under the impact of the high-energy electron beam. Consequently, the size of IONFs determined using HAADF-STEM ($D_P^{\text {STEM}}$) is smaller than the hydrodynamic diameter ($D_h^{\text {DLS}}$) determined using DLS^37^.

The mean sizes $\langle D^{\textrm{DLS}}_h\rangle$ and $\langle D^{\textrm{STEM}}_P\rangle$ and their standard deviations ($\sigma$), which are summarized in Table 1, were determined from the obtained size distributions (Fig. 7) using

4 $$\begin{aligned} \langle D\rangle = \int _{D_{\text {min}}}^{D_{\text {max}}}Dq_0(D)\text {d}D \end{aligned}$$

and

5 $$\begin{aligned} \sigma =\sqrt{\int _{D_{\text {min}}}^{D_{\text {max}}}\left[ (D-\langle D\rangle )^2 q_0(D)\right] \text {d}D} \end{aligned}$$

The difference between the mean hydrodynamic diameter, $\langle D^{\textrm{DLS}}_h\rangle = (29\pm 8)$ nm, and the mean diameter of the IONFs determined by HAADF-STEM, $\langle D^{\textrm{STEM}}_P\rangle = (20\pm 4)$ nm, reveals an estimate of the mean thickness of the dextran shell ($\approx 5$ nm). The mean IONF size obtained from HAADF-STEM, $\langle D^{\textrm{STEM}}_P\rangle = (20\pm 4)$ nm, agrees very well with the mean IONF size obtained from HRTEM, $\langle D^{\textrm{HRTEM}}_P\rangle = (19\pm 4)$ nm. A good agreement was also achieved for the mean size of the cores, $\langle D_C\rangle$, determined using HAADF-STEM and HRTEM. Additionally, HRTEM revealed the size of the slightly twisted core fragments, $\langle D_F\rangle$, which was visible by XRD as the mean size of individual crystallites (Fig. 5). Note that $\langle D^{\textrm{XRD}}_F\rangle$ is slightly smaller than $\langle D^{\text {HRTEM}}_F\rangle$, because XRD recognizes mainly the undisturbed interior of the NCs, while their possibly defect-rich rim contributes rather to diffuse scattering than to the diffraction lines. Thus, the difference between $\langle D^{\text {HRTEM}}_F\rangle$ and $\langle D^{\text {XRD}}_F\rangle$ can be understood as a first estimate of the thickness of the disordered surface layer of the core fragments, which is approximately 1 nm. The ‘cluster size’ of approx. 16 nm obtained from XRD corresponds to the size of agglomerates of partially coherent twisted domains. Its value is between the size of the cores $\langle D_C\rangle$ and the size of the IONFs $\langle D_P\rangle$ (Table 1), which illustrates once more the crystallographic partial coherence of the cores within IONFs discussed above.

**Table 1:** Hydrodynamic diameter $\langle D_h\rangle$, particle diameter $\langle D_P\rangle$, core diameter $\langle D_C\rangle$ and diameter of the core fragments $\langle D_F\rangle$ as determined by DLS, low-magnification HAADF-STEM, HRTEM and XRD.

	$\langle D_h\rangle\,[\text{nm}]$	$\langle D_P\rangle\,[\text{nm}]$	$\langle D_C\rangle\,[\text{nm}]$	$\langle D_F\rangle\,[\text{nm}]$
DLS	$29\pm 8$	–	–	–
HAADF-STEM	–	$20\pm 4$	$15\pm 4$	–
HRTEM	–	$19\pm 4$	$13\pm 3$	$9\pm 3$
XRD	–	$16\pm 3$		$7\pm 1$

### Influence of the structure of the IONFs on their magnetic properties

**Figure 8:**
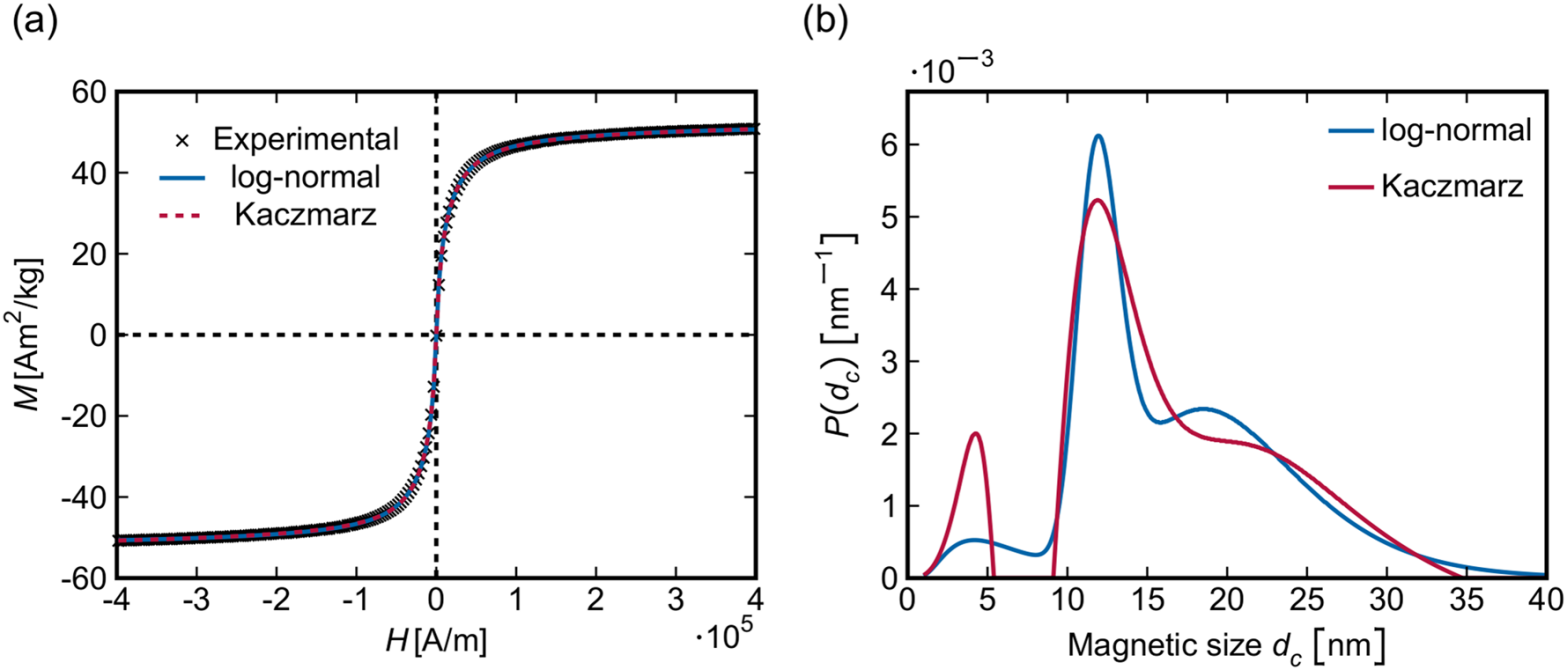
(**a**) Magnetization curve of the IONFs as measured by AGM (crosses) and the Langevin fits using three log-normal functions (solid blue line) and using the Kaczmarz method^70,71^ (dashed red line). (**b**) Distributions of the magnetic particle size corresponding to the fits in panel (**a**).

The magnetization curve of the IONFs measured by AGM and normalized to the sample density is depicted in Fig. 8a. The IONFs show superparamagnetic behavior that is characterized by negligible remanent magnetization and coercive field. The normalized (mass) saturation magnetization was ($50\pm 1$) Am$^2$/kg, which is lower than the saturation magnetization of bulk maghemite (74.3 Am$^2$/kg)^15^. Assuming that the saturation magnetization is reduced by the spin disorder in the surface layer of the magnetic particles, the ratio between the thickness of the disordered spin layer (*t*) and the particle size (*D*) can be calculated using the relation^24–26^

6 $$\begin{aligned} M_S = M_S^{\textrm{bulk}} \left( 1 - \frac{6t}{D}\right) \end{aligned}$$

For $M_S = (50\pm 1)$ Am$^2$/kg and $M_S^{\textrm{bulk}} = 74.3$ Am$^2$/kg, *t*/*D* is $(0.055 \pm 0.001)$. A disordered spin layer having a thickness of 1 nm^25^ would be consistent with a particle size of 18 nm, which agrees best with $\langle D_P\rangle$ from Table 1. A disordered spin layer having a thickness of 0.54 nm^26^ would correspond to a particle size of 10 nm, which is between $\langle D_F\rangle$ and $\langle D_C\rangle$.

For modelling of the measured magnetization curve, two approaches were used. Both are based on the approximation of the *M*(*H*) dependence by the Langevin function:

7 $$\begin{aligned} M(H) = M_S{\mathcal {L}}(\xi ) \end{aligned}$$

where $M_S$ is the saturation magnetization and ${\mathcal {L}}(\xi ) = \coth (\xi ) - 1/\xi$. The parameter $\xi$ is related to the (volume) saturation magnetization ($M_S$), to the strength of the external magnetic field (*H*), to the permeability of vacuum ($\mu _0$), to the Boltzmann constant ($k_B$) and to the sample temperature (*T*)^71,72^:

8 $$\begin{aligned} \xi (H) = \frac{M_S\pi d_c^3H\mu _0}{6k_BT} \end{aligned}$$

Note that in Eq. (8), $M_S$ has the unit of A/m like *H*. As the recorded signal is a superposition of the magnetizations of all particles in the sample, the size distribution of the magnetic particles must be taken into account.

In the first modelling approach, it was assumed in analogy to previous reports^33,35,72–75^ that the size distribution can be described by log-normal functions. As microstructure analyses revealed the existence of three different types of magnetic ‘objects’ (Table 1), a sum of three log-normal functions was employed for the Langevin fit:

9 $$\begin{aligned} P(d_c) = \sum _{i=1}^3w_i\frac{1}{\sqrt{2\pi }\sigma _i d_c}\exp \left[ -\frac{\left( \ln d_c - \mu _i\right) ^2}{2\sigma _i^2}\right] \end{aligned}$$

The refinable parameters were the weights of the log-normal functions ($w_i$), the medians of the magnetic particle sizes ($\mu _i$) and the widths of the log-normal functions ($\sigma _i$). The fitting function based on Eq. (7) had the form:

10 $$\begin{aligned} M(H) = M_S\int _0^\infty P(d_c){\mathcal {L}}(\xi )\text {d}d_c \end{aligned}$$

The﻿ best fit of the magnetization function (Fig. 8a) was obtained for the sizes of magnetic particles of ($6\pm 4$) nm, ($12\pm 1$) nm and ($20\pm 5$) nm, which agree well with the size of the fragments ($D^{\textrm{HRTEM}}_F$ and $D^{\textrm{XRD}}_F$), with the size of the cores ($D^{\textrm{STEM}}_C$ and $D^{\textrm{HRTEM}}_C$) and with the size of the IONFs ($D^{\textrm{STEM}}_P$ and $D^{\textrm{HRTEM}}_P$) from Table 1, respectively. The resulting size distribution function obtained from the Langevin fit is depicted in Fig. 8b. The sizes of very small particles (fragments of the cores and the cores themselves) determined from the magnetization curve are slightly smaller than the corresponding sizes $D_F$ and $D_C$ determined using HAADF-STEM, HRTEM and XRD as expected, because the magnetization of small particles is reduced by a disordered spin layer at their surface^24–26^.

In the second approach, the particle size distribution was substantially less constrained, as the shape of the distribution was determined using Kaczmarz’ iterative method^70,71^ without any a priori assumption (except keeping the values of the distribution function non-negative). Within this method, a matrix ${\textbf{A}}_{ji}$ is composed, which contains magnetization values calculated according to Eqs. (7) and (8) for individual values of the magnetic particle size ($d_{c,i}$) and for individual values of the external magnetic field ($H_j$). This matrix is used for iterative calculation of the ‘weighting factors’ *W*:

11 $$\begin{aligned} W^{k+1} = W^k +\frac{M_j-{\textbf{A}}_j W^k}{\Vert {\textbf{A}}_j\Vert ^2}{\textbf{A}}_j^\intercal \end{aligned}$$

that describe the particle size distribution. In Eq. (11), *k* is the iteration number. The starting set of the ‘weighting factors’ ($W^0$) is a zero vector having the same length like the vector $d_{c,i}$. $M_j$ are the magnetization values measured at different intensities of the external magnetic field $H_j$, and ${\textbf{A}}_j$ the corresponding row vectors of the ${\textbf{A}}_{ji}$ matrix (calculated for the same magnetic field $H_j$ but for different particle sizes $d_{c,i}$). After each iteration, negative values of *W* are reset to zero. Following previous reports^70,71^, 10,000 iterations were employed. The final fit of the magnetization curve obtained from

12 $$\begin{aligned} M_j^{\mathrm{(calc)}} = \sum _i W_i^{10,000}{\textbf{A}}_{ji} \end{aligned}$$

is depicted in Fig. 8a, the size distribution ($P(d_c)\,\widehat{=}\,W^{10,000}$) in Fig. 8b.

It can be seen from Fig. 8a that both approaches, which are the Langevin fit with three log-normal functions corresponding to the size distributions of the whole particles (IONFs), their cores and fragments, and the Langevin fit using Kaczmarz’ method, reveal almost the same magnetization curve despite the relatively large differences in the corresponding size distribution. This shows a relatively low sensitivity of the magnetization curve to the exact particle size distribution and suggests that additional information obtained from structure analysis, e.g., information about the number of different magnetic objects, can help to improve the reliability of the size distribution.

## Discussion

**Figure 9:**
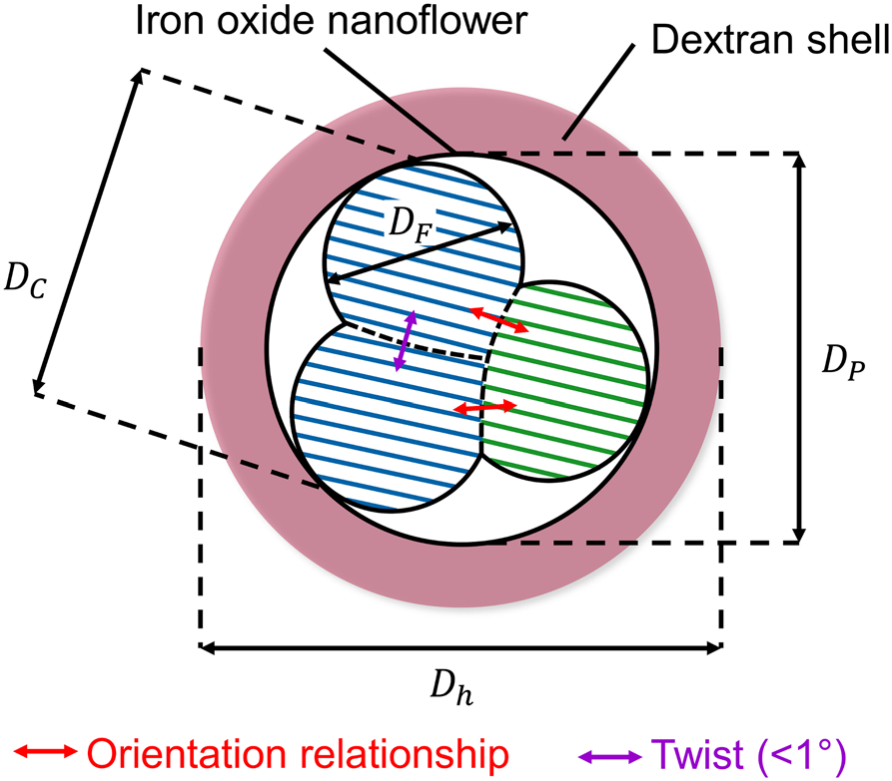
Schematic illustration of the hierarchical structure of a dextran-coated IONF, adapted from Gavilán et al.^35^ and modified. Hydrodynamic diameter $D_h$, particle diameter $D_P$, core diameter $D_C$ and diameter of the core fragments $D_F$ are indicated. Red and purple arrows mark neighboring cores with lattice planes with matching interplanar spacings and fragmented cores with nearly identical crystallographic orientation, respectively.

In analogy with the paper from Gavilán et al.^35^, where a hierarchical structure of similarly synthesized IONFs was characterized and described by a multimodal size distribution, the IONFs under study were found to be composed of agglomerated maghemite NCs (Fig. 9). Our XRD and HRTEM analyzes identified the NCs as elementary blocks forming the magnetic cores and IONFs. The mean sizes of the NCs were $\langle D^{\text {XRD}}_F \rangle = (7 \pm 1)$ nm and $\langle D^{\text {HRTEM}}_F \rangle = (9 \pm 3)$ nm, cf. Table 1. The difference in the size of the core fragments obtained from XRD and HRTEM is connected with a different sensitivity of the analytical techniques to the structural disorder at the surface of the NCs. XRD recognizes only the coherent part of the NCs as the core fragments. Therefore, it reveals the size of their undisturbed interior, while HRTEM sees the core fragments including their rim, in particular for isolated NCs. The NCs were also recognized by the Langevin fit of the magnetization curve. Their ‘magnetic’ size was $(6 \pm 4)$ nm. The amount of the NCs determined from the magnetic measurement was relatively low (Fig. 8b), because the majority of neighboring NCs possessed almost the same crystallographic orientation, as revealed by HRTEM (Fig. 2b) and as concluded from the coherence phenomena affecting the XRD line broadening (Fig. 5). The misorientation of the NCs within the cores was below $1^{\circ }$, as revealed by GPA of the HRTEM images (Fig. 4) and by XRD (Fig. 5). This kind of crystallographic coherence facilitates coupling of magnetic moments in individual NCs forming the cores^33,42^. Thus, the magnetic measurement recognized much more cores than isolated NCs (Fig. 8).

The size of the cores can be determined most reliably using HRTEM in combination with local orientation analysis (FFT/HRTEM or GPA). HAADF-STEM may overestimate the size of the cores, because it uses a shape-based segmentation routine to identify individual cores in the IONFs (Fig. 6). However, this routine cannot distinguish parts of the IONFs with different crystallographic orientations from each other like HRTEM complemented by FFT or GPA. XRD can only estimate the size of the cores from the size of the clusters composed of partially coherent NCs (core fragments). The ‘magnetic’ size of the cores, $\langle D^{\text {AGM}}_C \rangle = (12 \pm 1)$ nm, refers to the size of magnetic domains with uniform orientation of spin moments. Thus, half of the difference between $\langle D^{\text {HRTEM}}_C \rangle = (13 \pm 3)$ nm and $\langle D^{\text {AGM}}_C \rangle$ can be understood as the thickness of the disordered spin layer of the cores. According to Eq. (6), a disordered spin layer having a thickness of $\approx 0.5$ nm would reduce the saturation magnetization of the cores from 74.3^15^ to 57.1 Am$^2$/kg, which approaches the saturation magnetization of 50 Am$^2$/kg obtained from the Langevin fit of the magnetization curve (Fig. 8). Note that Sharifi Dehsari et al.^26^ reported about a disordered spin layer having a thickness of 0.54 nm. As reported by Morales et al.^22^, an additional reason for the reduction of the saturation magnetization might be a certain degree of disorder of the spins even in the volume of the IONFs as a result of an inhomogeneous ordering of the cation vacancies in the IONFs (Fig. 2d).

A large part of the cores in the IONFs possessed distinct mutual crystallographic orientation relationships (Figs. 3 and 4), which resulted from their attachment along lattice planes with matching interplanar spacings. The attachment of the cores along lattice planes with the same interplanar spacing is a phenomenon, which was observed even in dual-phase systems with different crystal structures of the counterparts^76^. Such cores are not mutually coherent for XRD, and can be easily distinguished by FFT/HRTEM because of their different crystallographic orientations. In contrast to XRD and HRTEM, low-magnification HAADF-STEM cannot distinguish these two kinds of cores from each other directly, but it identifies these cores just as convex parts of the IONFs. Furthermore, it should be mentioned that the determination of the size of the cores from low-magnification HAADF-STEM images does not succeed, when the cores overlap in the projection direction. However, this was rarely the case in our IONFs.

The IONFs under study are agglomerates of cores consisting of individual NCs. The size of the IONFs was quantified using both, HRTEM and HAADF-STEM (Table 1). Still, low-magnification HAADF-STEM is more reliable than HRTEM from the statistical point of view, because it allows more IONFs to be analyzed (Fig. 6). The accuracy of low-magnification HAADF-STEM for the determination of the size of the IONFs is sufficient, as only one segmentation step, i.e., the semi-automatic segmentation based on a marker-based watershed algorithm, is required^45,66^. From the point of view of the magnetic properties, the IONFs can behave as magnetic particles with uniform orientation of magnetic moments, even if their cores are crystallographically non-coherent. Still, adjacent cores should be attached along specific lattice planes like in Figs. 3 and 4, and the angle between the easy magnetization axes of the individual cores should be small. Therefore, a cooperative magnetic behavior is expected also within the multi-core IONFs. A magnetic coupling was confirmed by the presence of magnetic particles having a size of $(20 \pm 5)$ nm as concluded from the Langevin fit of the magnetization curve (Fig. 8). This particle size agrees very well with the size of the IONFs, which was $(20 \pm 4)$ nm and $(19 \pm 4)$ nm according to HAADF-STEM and HRTEM, respectively.

The structure of the IONFs under study can be summarized as follows. The IONFs with the size $D_P$ are composed of several cores having the size $D_C$ (Fig. 9). The cores consist of several NCs having the size $D_F$. Individual NCs contain maghemite with the average chemical composition $\gamma$-Fe$_{2.72\pm 0.02}$O$_4$ and with partially ordered vacancies on metallic positions (Fig. 2d). The main driving force for the clustering of NCs and for the formation of the cores and IONFs is the minimization of the surface energy *via* oriented attachment of primary NCs along certain crystallographic facets^33,40–43^. This mechanism generally involves rotations of the NCs in three-dimensional space, until they share the same facets^77^. However, this process depends strongly on the reaction conditions. It has been shown previously that the internal structure of IONFs is influenced by many different parameters of the synthesis process, e.g., by the nature of the polyol solvent^41,43^, by the heating temperature, heating time and heating rate^38,39,78^, by the stoichiometry of the iron precursor^10,39^ and by the presence and concentration of a reducing agent^32,41,78^. The arrangement of the cores in IONFs is controlled primarily by the kinetics of the nucleation and aggregation of the primary NCs, which in turn depends on the type of polyol used for the synthesis^43^. Higher formation and growth rates of the NCs cause a faster aggregation resulting in a higher misalignment of the NCs within the IONFs. As we observed not only a fully epitaxial alignment but also specific orientation relationships between individual NCs building up the IONFs, we can conclude that the nucleation and aggregation of the NCs in our IONFs was slightly too fast. Consequently, not all NCs did have enough time to order to possess the same crystallographic orientation. Some NCs were just oriented along specific lattice planes that were parallel to each other. This kind of alignment of NCs might partially reduce the surface energy but also inhibit a full alignment of the NCs. Moreover, this alignment of NCs produces local strain fields, which are compensated by crystal structure defects, possibly dislocations (Fig. 3).

## Conclusions

A combination of TEM, XRD and DLS disclosed the hierarchical architecture of dextran-coated multi-core IONFs prepared by a polyol method. The TEM measurements combined high-resolution (HRTEM with FFT and GPA) and low-resolution (HAADF-STEM) modes in a correlative multiscale approach in order to describe the internal structure of the IONFs on the atomic scale including the orientation relationships between individual NCs and cores, and to determine the size distribution of the constituents in a statistically relevant manner. It was shown that the basic units of the IONFs are maghemite NCs with partially ordered vacancies on the iron sites. NCs with distinct crystallographic orientation relationships form magnetic cores, which agglomerate and build up the IONFs. Neighboring cores were typically attached by sharing lattice planes with the same interplanar distance. The presence of these objects was confirmed by the Langevin fit of the magnetization curve measured using AGM. As the magnetic sizes of the NCs, of the cores and of the IONFs were very close to the corresponding sizes obtained from the microstructure analysis, it was concluded that the magnetic moments of individual NCs interact mutually. It was shown that the magnetic interaction between individual NCs and cores is strongly affected by their mutual crystallographic orientation. The strongest coupling of magnetic moments was observed between neighboring NCs that had almost the same crystallographic orientation and that formed the magnetic cores. A weaker but still existing magnetic interaction was detected between the magnetic cores within individual IONFs, which had a distinct orientation relationship but no full crystallographic coherence. From the difference between the particle sizes obtained from the microstructure analysis and from the magnetic measurement, it was concluded that the magnetic cores have a disordered spin layer at the rim. This layer, which has a thickness of approximately 0.5 nm, reduces the saturation magnetization of the IONFs together with the inhomogeneous ordering of the vacancies on the iron sites in $\gamma$-Fe$_{2.72\pm 0.02}$O$_4$.

## Data Availability

The datasets analyzed in the current study are available from the corresponding author on request.
